# Acute pericarditis complicated with pericardial effusion as first presentation of COVID‐19 in an adult sudanese patient: A case report

**DOI:** 10.1002/ccr3.5570

**Published:** 2022-03-22

**Authors:** Waddah Aljaely Mohamed Osman, Abdelmuniem Siddig Mohamed Ahmed, Moh. Mah. Fadelallah Eljack, Khabab Abbasher Hussien Mohamed Ahmed, Mazin S. Haroun, Yassin Abdelrahim Abdalla

**Affiliations:** ^1^ Faculty of Medicine University of Gezira Wad Medani Sudan; ^2^ Faculty of Medicine, Physiology department Wad Medani Teaching Hospital University of Gezira Wad Medani Sudan; ^3^ Faculty of Medicine Medani Heart Centre University of BakhtAlruda Wad Medani Sudan; ^4^ Faculty of Medicine University of Khartoum Khartoum Sudan; ^5^ Faculty of Medicine University of Khartoum Khartoum Sudan; ^6^ Department of Internal Medicine Faculty of Medicine and Health Sciences Omdurman Islamic University Khartoum Sudan

**Keywords:** COVID‐19, pericardial effusion, pericarditis, sudan

## Abstract

Acute pericarditis is an uncommon presentation of COVID‐19. Here, we described a case of a 50‐year‐old male patient who presented with chest pain without fever or cough and diagnosed with acute pericarditis complicated by pericardial effusion due to COVID‐19 after exclusion of other causes and received supportive treatment and improved over two weeks.

## Graphical abstract

Although it is a rare presentation, suspicion of COVID‐19 infection in patients with acute pericarditis is crucial in the setting of pandemic irrespective of the absence of a typical clinical feature of COVID‐19.

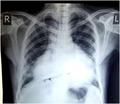

## 1 INTRODUCTION

Since the first case of coronavirus disease (COVID‐19) in Wuhan, China, in late December 2019 and up to now with emerging of mutated virus variants such as Delta and Omicron variants; severe acute respiratory syndrome coronavirus‐2 (SARS‐CoV‐2) remains the most common presentation, which frequently presents with respiratory symptoms that can progress to pneumonia, and in severe cases, acute respiratory distress syndrome (ARDS) and shock.^1^ However, there are increasing concerns of cardiovascular manifestations and complications that can occur due to the virus, which may occur either isolated or concomitant with a pulmonary disease. The commonest cardiac complications include acute myocardial injury, arrhythmias, acute myocarditis, and severe left ventricular dysfunction.^2^ Conversely, some cases of pericarditis and pericardial effusion have been reported within recent months, which necessitate this case report to review and improve understanding of uncommon presentations and complications that may occur with COVID‐19.^3^ Hereby, we report a 50‐year‐old male patient who presented with acute chest pain diagnosed as acute pericarditis complicated with pericardial effusion due to COVID‐19 after exclusion of other possible causes.

## 2 CASE PRESENTATION

A previously healthy 50‐year‐old Sudanese man with a clear medical background presented to Medani Teaching Hospital in Wad Medani, Sudan, complaining of chest pain; stabbing in nature, increased in severity in supine position and improved by leaning forward. He denied any history of cough or shortness of breath.

During physical examination, the patient noticed that he preferred sitting leaning forward, but not dyspneic. His BP was 160/80, pulse rate 96 regular, temperature 38.1 and oxygen saturation 96% on room air. The JVP was not raised. Pericardial friction rub was not heard. Cardiovascular and chest examination was unremarkable.

Initial laboratory investigations revealed the following: WBCs 11.0 × 109/L (4.0 −11.0 × 109/L), lymphopenia 5%, ESR 50 mm/hour, the liver and kidney function were within normal values. D‐dimer was 350 ng/ml. Serum quantitative troponin was 0.5 ng/ml (normal laboratory value 0–0.6 ng/ml). ECG showed widespread ST‐segment elevation, and the baseline transthoracic echocardiography was normal; hence, the patient was diagnosed as acute pericarditis according to European Society of Cardiology Diagnostic Criteria (Typical pericardial chest pain and new widespread ST‐segment elevation) in addition to raised inflammatory markers. At first, the patient received I.V fluids, ibuprofen tablets 600 mg TDS and was advised to restrict exercise, but the presence of lymphopenia necessitated exclusion of COVID‐19—as an emerging pandemic—so nasopharyngeal swabs had been taken for (PCR), which came back later positive. The patient was admitted to the isolation center of COVID‐19. According to local guidelines of COVID‐19 management, the patient was started on paracetamol infusion, low molecular weight heparin (LMWH) 75 IU/kg, dexamethasone 6 mg QID, and vitamin supplement, and Ibuprofen tabs were replaced with paracetamol.

**FIGURE 1:**
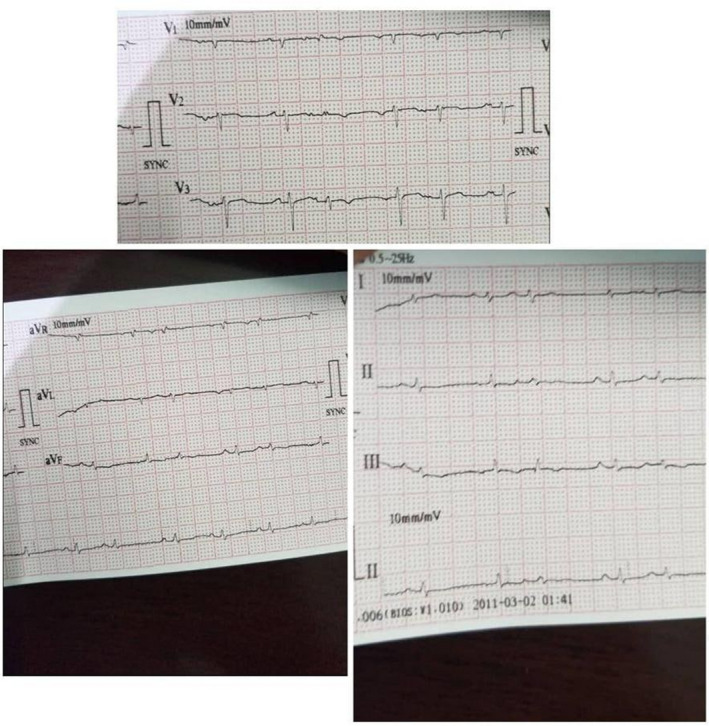
Electrocardiography (ECG): It showed low voltage in all leads

**FIGURE 2:**
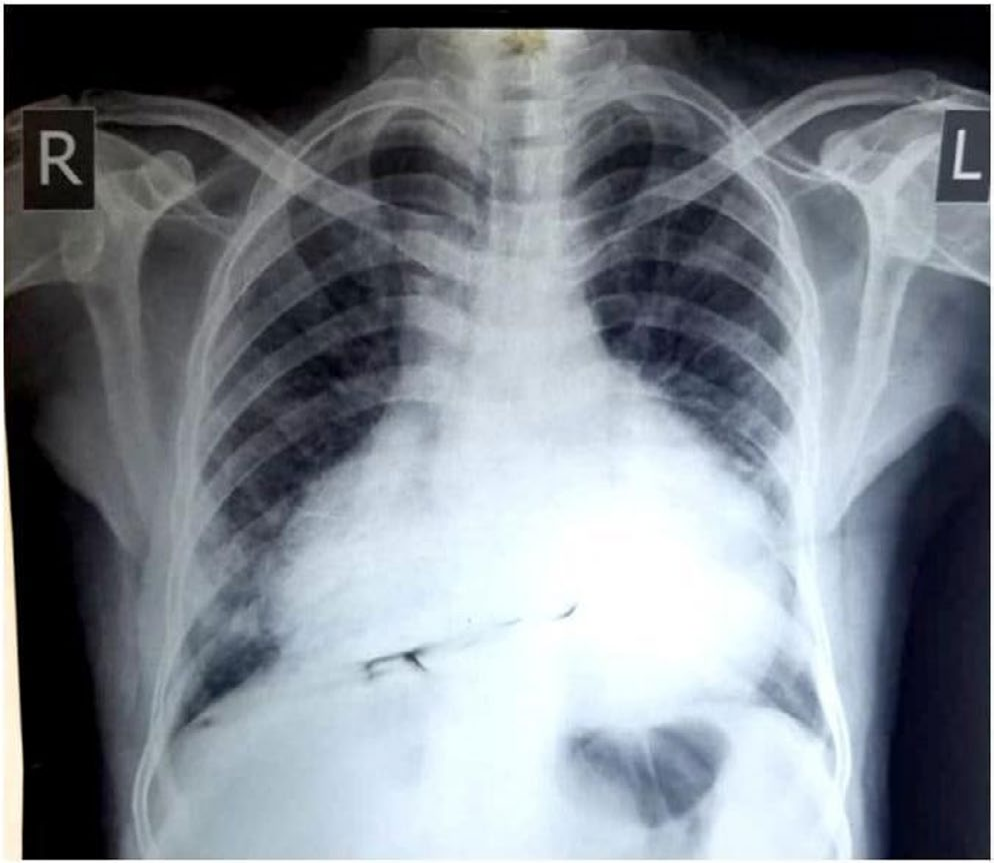
Chest X‐ray: It showed enlargement of cardiac outline with the absence of pulmonary infiltratesreference

On the fifth day of admission, the patient developed mild shortness of breath and dry cough. Physical examination revealed a dyspneic, tachypneic patient without raised JVP; BP was 140/80; further assessment showed that the apex beat was difficult to locate with muffled first and second heart sounds; also, there were no added sounds or murmurs. The ECG showed low voltage QRS complexes in all leads (Figure 1). The chest radiograph revealed an enlarged cardiac silhouette with absence of pulmonary infiltrates, and no pleural effusion was detected (Figure 2). Subsequently, a transthoracic echocardiogram was performed which showed a moderate pericardial effusion not consisting of tamponade, with no regional wall motion abnormalities. Left ventricular ejection fraction was 55%. Laboratory investigations showed an elevated C‐reactive protein (CRP) level 150 mg/L (0–10 mg/L). Pericardiocentesis was not performed considering the absence of cardiac tamponade features, moderate pericardial effusion, and that there was no suspicion of bacterial or neoplastic etiology. The patient was continued on LMWH 75 IU/kg, dexamethasone 6 mg QID, and vitamin supplements; follow‐up 8 days later showed clinical and laboratory improvement, and subsequent resolution of effusion on echocardiogram only to confirm COVID‐19 clearance with a negative PCR, which made discharge a suitable decision, and for the patient to be seen one month later on refer clinic.

## 3 DISCUSSION

The coronavirus infection 2019 (COVID‐19), which is caused by SARS‐CoV‐2, causes mainly respiratory tract symptoms. While much of the focus has been on pulmonary complications, emergency clinicians need to be aware of the cardiovascular complications as well, which can be a significant contributor to the deaths associated with this disease. Cardiovascular complications that are associated with COVID‐19 include arrhythmias, acute myocardial infarction, myocarditis, heart failure, and thromboembolic events.^4^ These complications could be explained by many mechanisms such as plaque rupture and coronary thrombosis, exaggerated immune response, direct myocardial injury, systemic inflammation, altered myocardial demand‐supply ratio, electrolyte imbalances, or adverse effects of therapies.^5^

Acute pericarditis, which is an inflammation of the sac surrounding the heart, is a rare extra‐pulmonary complication of COVID‐19. Generally, the etiology of pericarditis could be classified into infectious and non‐infectious.^6^ Both depend on the clinical context and epidemiological framework; in developing countries, infectious etiologies represent the most common causes, whereas idiopathic or post‐viral infection are the major causes of this illness in developed countries. Tuberculosis is the commonest cause globally.^1^ Generally, it is postulated that viruses can cause pericardial inflammation via direct cytotoxic effects or immune‐mediated mechanisms.^7^ COVID‐19 has been stated to trigger an exaggerated systemic inflammatory response in certain patients.^8^ Likewise in other viral infections, this inflammatory response could lead to pericarditis and the consequent pericardial effusion; however, the exact mechanism is unknown. According to European Society of Cardiology (ESC) guidelines, the diagnosis of acute pericarditis depends on the presence of at least two out of four criteria: pericarditis chest pain, pericardial rub, new widespread ST‐segment elevation or PR depression, and new or worsening pericardial effusion. Additional finding that can support the diagnosis of acute pericarditis includes elevation of inflammatory markers (white blood cell count, C‐reactive protein, and erythrocyte sedimentation rate), evidence of pericardial inflammation on computed tomography (CT) and cardiac magnetic resonance (CMR).

However, in our patients, we did not perform pericardiocentesis due to the nonexistence of cardiac tamponade features, the moderate pericardial effusion, and absence of suspicion regarding bacterial or neoplastic etiology. Moreover, the molecular diagnosis of other viral causes of pericarditis is unavailable in our center. Therefore, the diagnosis of pericarditis due to COVID‐19 in this patient is presumptive.

The mainstay treatment of acute pericarditis is a high‐dose aspirin and NSAIDs; other options include colchicine.^7^ A patient who has contraindications or failed to respond to first‐line therapy may benefit from corticosteroids. In our patient, we considered administering colchicine—as it remains a more suitable drug than steroids—but it was unavailable in the center. Using corticosteroids and NSAIDs could worsen the general condition of COVID‐19 patients, and this has emerged as a management dilemma due to safety concerns regarding our patient.^7^ To date, no evidence prohibits the use of high‐dose aspirin in COVID‐19 patients.^5^ The matters were mainly regarding the use of ibuprofen, and there is no compelling evidence available connecting “worsening of COVID‐19 patients’ status” with “aspirin or other NSAIDs.” To navigate this uncertainty, the use of NSAIDs when clinically indicated was supported by the Centre for Disease Control and Prevention (CDC) and the US Food and Drug Administration (FDA).

The most frequent anticoagulant for COVID‐19 patients is LMWH, which is correlated with survival when administered at a prophylactic dose.^9^ Regarding use of LMWH in our patient, we continued its administration until the patient showed improvement and clearance of COVID‐19 hence discharge.

Predictors of poor prognosis in acute pericarditis include the following: fever more than 38, large pericardial effusion, sub‐acute onset, cardiac tamponade, and lack of response to NSAID or aspirin after at least one week of therapy.^10^ Our patient presented with a fever of 38.1 C, which resembled a poor prognostic feature, hence required additional monitoring; but due to the preparedness of the team and the careful evaluation, we were fortunate to tackle this case successfully. Therefore, in order to achieve the best outcome for patients, we emphasize on the importance of prior knowledge of prognostic factors, careful evaluation, and immediate optimal responses.

## 4 CONCLUSION

Acute pericarditis and pericardial effusion are rare extra‐pulmonary presentations of COVID‐19, especially without concomitant pulmonary disease or myocardial injury. A high index of suspicion is necessary to assure early diagnosis and treatment. Studies are needed to determine the exact mechanism of acute pericarditis and pericardial effusion in COVID‐19 patients and the optimal therapeutic strategies.

## CONFLICT OF INTEREST

The authors declare that there is no conflict of interest.

## AUTHOR CONTRIBUTION

WAM, ASM, and MMF collected data and did investigations. KAH, MSH, and YAA wrote and revised the first and final drafts. All authors contributed significantly in all stages of the manuscript.

## CONSENT

Written consent for publication has been obtained from the patient and the authors.

## Data Availability

The data used in this report are available with the corresponding author upon reasonable request.
